# Preoperative chemotherapy with docetaxel, oxaliplatin, and S-1 for gastric cancer with extensive lymph node metastasis: 3-year follow-up results from JCOG1704

**DOI:** 10.1007/s10120-026-01766-3

**Published:** 2026-06-10

**Authors:** Yukinori Kurokawa, Yuichiro Doki, Junki Mizusawa, Kota Kawabata, Takashi Nomura, Kunihiro Tsuji, Sang-Woong Lee, Haruhiko Cho, Jun Hihara, Naoki Hiki, Souya Nunobe, Narikazu Boku, Masanori Terashima, Takaki Yoshikawa

**Affiliations:** 1 Department of Gastroenterological Surgery, The University of Osaka Graduate School of Medicine, 2-2-E2, Yamadaoka, Suita, Osaka, 565-0871 Japan; 2 Japan Clinical Oncology Group Data Center/Operations Office, National Cancer Center Hospital, Tokyo, Japan; 3 Department of Surgery, Yamagata Prefectural Central Hospital, Yamagata, Japan; 4 Department of Medical Oncology, Ishikawa Prefectural Central Hospital, Kanazawa, Japan; 5 Department of General and Gastroenterological Surgery, Osaka Medical and Pharmaceutical University Hospital, Osaka, Japan; 6 Department of Surgery, Tokyo Metropolitan Cancer and Infectious Diseases Center Komagome Hospital, Tokyo, Japan; 7 Department of Surgery, Hiroshima City North Medical Center Asa Citizens Hospital, Hiroshima, Japan; 8 Department of Upper Gastrointestinal Surgery, Kitasato University School of Medicine, Kanagawa, Japan; 9 Department of Gastroenterological Surgery, Cancer Institute Hospital, Japanese Foundation for Cancer Research, Tokyo, Japan; 10 Department of Oncology and General Medicine, Institute of Medical Science, IMSUT Hospital, University of Tokyo, Tokyo, Japan; 11 Division of Gastric Surgery, Shizuoka Cancer Center, Nagaizumi, Japan; 12 Department of Gastric Surgery, National Cancer Center Hospital, Tokyo, Japan

**Keywords:** Neoadjuvant DOS chemotherapy, Perioperative treatment, Bulky node metastases, Para-aortic node metastases, Oligometastases

## Abstract

**Background:**

Prognosis of marginally resectable gastric cancer with extensive lymph node metastasis (ELM) remains poor, even after R0 resection. JCOG0405 established preoperative cisplatin plus S-1 as a provisional standard of care in Japanese guidelines, based on a favorable 3-year overall survival (OS) of 58.8%. We report the 3-year survival outcomes of JCOG1704, a phase II trial evaluating preoperative docetaxel, oxaliplatin, and S-1 (DOS) for marginally resectable gastric cancer with ELM, following promising short-term outcomes.

**Methods:**

Patients with histologically confirmed HER2-negative gastric adenocarcinoma and bulky nodal (BN) involvement and/or para-aortic node (PAN) metastasis were eligible. Patients received three cycles of DOS (docetaxel 40 mg/m^2^, day 1; oxaliplatin 100 mg/m^2^, day 1; S-1 80–120 mg/m^2^, days 1–14) every 3 weeks, followed by gastrectomy with D2 plus PAN dissection and postoperative S-1 for 1 year.

**Results:**

Between December 2018 and March 2022, 47 patients (BN only, 20; PAN only, 17; both, 10) were enrolled. At the data cutoff date (May 2025), the 3-year OS and progression-free survival (PFS) were 86.7% (95% CI 72.7–93.8%) and 75.6% (95% CI 60.2–85.6%), respectively, among 46 eligible patients. Among 42 patients who underwent R0 resection, the 3-year relapse-free survival (RFS) was 78.6% (95% CI 62.9–88.2%). In subgroup analyses, the 3-year OS/PFS/RFS were 85.0%/75.0%/77.8% in BN only, 93.8%/81.3%/86.7% in PAN only, and 77.8%/66.7%/66.7% in both.

**Conclusions:**

Preoperative DOS for gastric cancer with ELM yielded unprecedentedly favorable 3-year survival outcomes and was considered a provisional standard of care without a phase III trial within our JCOG community.

## Introduction

Gastric cancer, the fifth leading cause of cancer-related deaths worldwide [1], has the highest incidence and mortality rates among all gastroenterological malignancies in Japan [2]. Surgical resection remains the only curative treatment for gastric cancer [3, 4], although some patients present with bulky lymph node metastases along main feeding arteries or para-aortic lymph node metastases. The prognosis of patients with gastric cancer accompanied by extensive lymph node metastasis (ELM) is extremely poor, even after R0 resection. Since 2000, the Stomach Cancer Study Group (SCSG) of the Japan Clinical Oncology Group (JCOG) has conducted a series of phase II trials, namely, JCOG0001, JCOG0405, and JCOG1002, to evaluate preoperative chemotherapy and improve outcomes in these patients with marginally resectable cancer [5–7]. In JCOG0405, preoperative cisplatin plus S-1 achieved a favorable 3-year overall survival (OS) of 58.8%, albeit with a low pathological complete response (pCR) rate of 2%. Based on these results, the Japanese Gastric Cancer Treatment Guidelines currently recommend preoperative chemotherapy with cisplatin plus S-1 followed by postoperative S-1 as the provisional standard perioperative treatment for gastric cancer with ELM [8].

A recent Korean phase III trial (PRODIGY) demonstrated significant improvements in progression-free survival (PFS) and OS with preoperative chemotherapy using docetaxel, oxaliplatin, and S-1 (DOS) followed by postoperative S-1 compared with upfront surgery followed by postoperative S-1 in patients with cT2–3N1 or cT4 gastric cancer [9, 10] One study from The University of Osaka in Japan reported the favorable efficacy and safety of preoperative DOS in a phase II trial, in which the docetaxel dose was reduced from 50 mg/m^2^ used in the PRODIGY trial to 40 mg/m^2^, for patients with cStage III gastric or esophagogastric junction adenocarcinoma [11]. Based on these findings, the SCSG/JCOG initiated JCOG1704, a phase II trial, in 2018 to evaluate the efficacy and safety of preoperative DOS, *i.e.*, the University of Osaka regimen, in gastric cancer patients with ELM [12]. We previously reported promising short-term outcomes, including an R0 resection rate exceeding 90% in this marginally resectable population [13]. Moreover, the major pathological response (grade ≥ 2, according to the Japanese Classification of Gastric Carcinoma [JCGC]) and pCR rates were 57% (26/46) and 24% (11/46), respectively. Here, we report the 3-year follow-up survival results of JCOG1704.

## Patients and methods

### Patients

JCOG1704 was a phase II multicenter trial conducted in 22 institutions, and the detailed inclusion and exclusion criteria were reported previously [12]. Briefly, eligible patients had histologically confirmed HER2-negative gastric adenocarcinoma with bulky nodal (BN) involvement at stations 7, 8a, 9, 11, 12a, or 14v, defined as one lymph node measuring ≥ 3 cm or two lymph nodes measuring ≥ 1.5 cm, and/or para-aortic nodal (PAN) involvement at stations 16a2 or 16b1, defined as a lymph node size of ≥ 1 cm on contrast-enhanced computed tomography. Additional eligibility criteria included an age of 20–75 years, an Eastern Cooperative Oncology Group performance status of 0 or 1, adequate oral intake, and sufficient organ function (neutrophil count, ≥ 2000/mm^3^; hemoglobin, ≥ 8.0 g/dL; platelet count, ≥ 100,000/mm^3^; serum total bilirubin, ≤ 1.5 mg/dL; serum aspartate aminotransferase, ≤ 100 IU/L; serum alanine aminotransferase, ≤ 100 IU/L; and creatinine clearance, ≥ 50 mL/min). Patients with distant metastases other than PAN metastasis, esophageal invasion of > 3 cm, remnant gastric cancer, large (≥ 8 cm) macroscopic type 3 tumors, or macroscopic type 4 tumors were excluded. In addition, patients with a history of gastric cancer surgery, except for bypass surgery or endoscopic resection, and those with a history of prior chemotherapy or radiotherapy for other malignancies were ineligible.

Before enrollment, the absence of peritoneal metastasis and negative peritoneal lavage cytology had to be confirmed by staging laparoscopy or laparotomy, and written informed consent was obtained from all patients. The study protocol was approved by the JCOG Protocol Review Committee and the Certified Review Board of the National Cancer Center Hospital. The trial was registered in the Japan Registry of Clinical Trials (jRCTs031180028).

### Procedures

Patients received three cycles of preoperative chemotherapy, including docetaxel (40 mg/m^2^) and oxaliplatin (100 mg/m^2^) on day 1 and S-1 (80 mg/m^2^/day) on days 1–14, repeated every 3 weeks. The detailed criteria for dose reduction and suspension of S-1 were previously reported [12].

Surgery was scheduled 4–8 weeks after the final administration of S-1, provided that no unresectable disease or inadequate organ function was identified. Patients underwent distal or total gastrectomy with D2 lymphadenectomy plus PAN dissection at stations 16a2 and 16b1. Spleen was preserved unless the tumor involved the greater curvature of the upper stomach or metastasis to the splenic hilar lymph nodes (station 10) was suspected. The reconstruction method was not specified. Laparoscopic or robotic gastrectomy was not permitted. In cases where unresectable disease, including those with positive peritoneal cytology, was intraoperatively detected, protocol treatment was discontinued.

Postoperative chemotherapy with S-1 (80 mg/m^2^/day) on days 1–28, repeated every 6 weeks, was initiated within 6 weeks after the R0 resection and continued for up to 1 year after surgery. Protocol treatment was discontinued in cases where postoperative S-1 could not be initiated within 12 weeks after surgery.

Patients were followed for at least 5 years after surgery. Serum carcinoembryonic antigen and carbohydrate antigen 19–9 levels were measured every 3 months for the first 3 years and every 6 months thereafter for 2 years. Contrast-enhanced abdominal computed tomography was performed every 6 months for the first 3 years and annually thereafter for 2 years. Upper gastrointestinal endoscopy was performed annually except in patients who had undergone total gastrectomy. Annual chest radiographies were also performed.

### Outcomes

The primary endpoint was major pathological response rate (pRR), defined as the proportion of patients who achieved JCGC grade 3 (absence of viable tumor cells), grade 2b (viable tumor cells < 1/10 of the tumor area), or grade 2a (viable tumor cells between 1/10 and 1/3 of the tumor area) response. Secondary endpoints included OS, relapse-free survival (RFS) in patients who underwent R0 resection, proportion of patients undergoing R0 resection, response rate to preoperative chemotherapy according to Response Evaluation Criteria in Solid Tumors (version 1.0), proportions of patients completing preoperative chemotherapy and protocol-specified surgery, proportions of patients completing all protocol treatments, and adverse events.

OS was defined as the time from enrollment to death from any cause. RFS was defined as the time from enrollment to first relapse or death from any cause. PFS was not prespecified as a secondary endpoint in the protocol. In this report, PFS was defined as the time from enrollment to disease progression or death from any cause. Disease progression during preoperative treatment was not considered a PFS event if preoperative treatment was followed by R0 or R1 resection. In patients who underwent R2 resection, radiologically confirmed progression after surgery was considered a PFS event. Adverse events were graded according to the Common Terminology Criteria for Adverse Events (version 4.0). Clinical and pathological staging was assessed according to the 15^th^ edition of the JCGC.

### Statistical analysis

In JCOG0405, which evaluated preoperative cisplatin plus S-1 by employing the same eligibility criteria, the major pRR was 27%. Using a two-stage design by the Southwest Oncology Group [14], assuming an expected major pRR of 40%, a threshold of 25%, a power of 80%, and a one-sided alpha level of 10%, the required sample size was 45. To account for potential ineligibility, the target enrollment was set to 50 patients. Confidence intervals were calculated using the Clopper–Pearson method. Time-to-event type endpoints were estimated using the Kaplan–Meier method. All analyses were performed using the SAS® software (version 9.4 or later; SAS Institute, Cary, NC, USA).

## Results

**Fig. 1:**
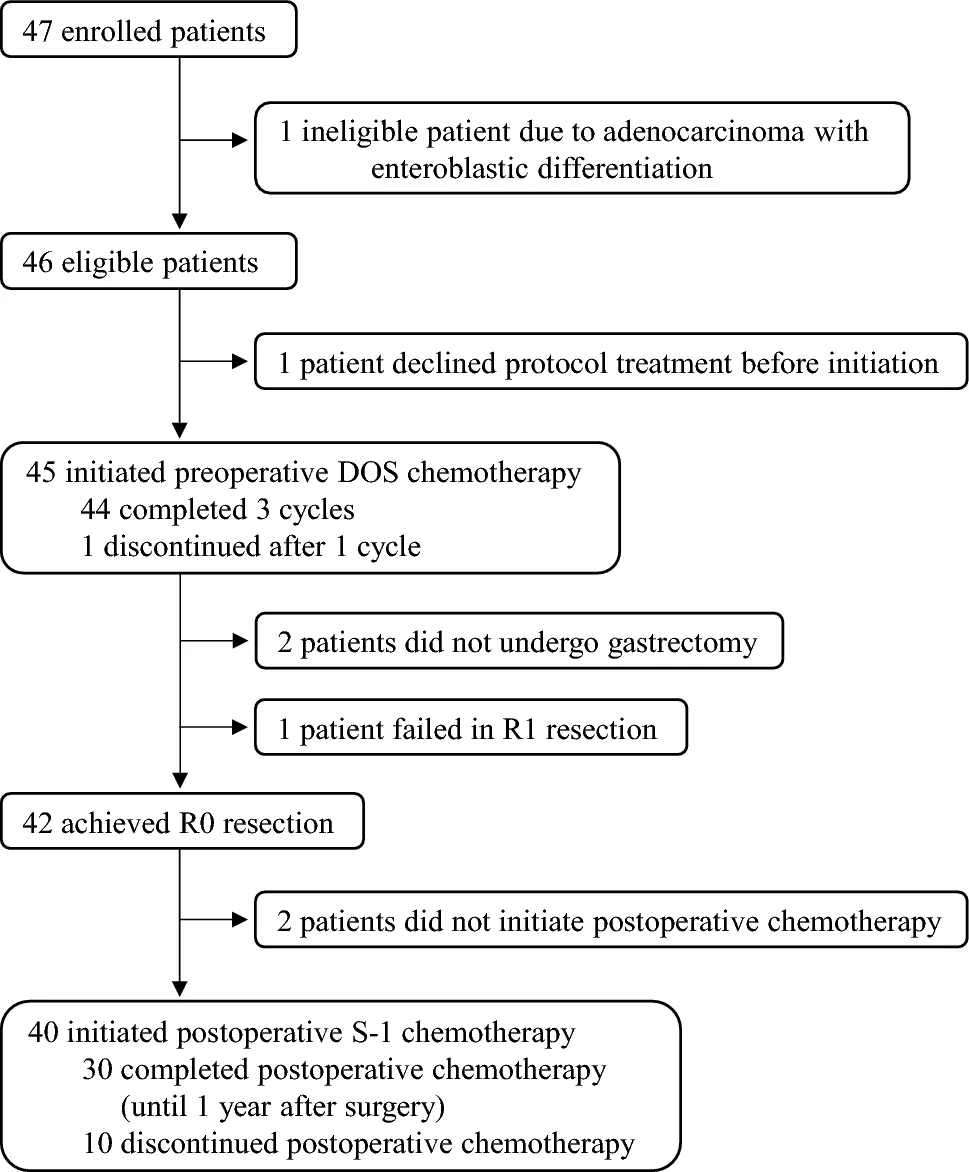
Study flow diagram

Between December 2018 and March 2022, 47 patients, including 20 patients with BN involvement only, 17 patients with PAN metastasis only, and 10 patients with both BN and PAN involvement, were enrolled in the trial (Fig. 1). One patient was deemed ineligible due to adenocarcinoma with enteroblastic differentiation. The baseline characteristics of the 46 eligible patients are summarized in Table 1. One eligible patient declined protocol treatment before initiation. Among the remaining 45 eligible patients who initiated preoperative DOS chemotherapy, 44 (97.7%) completed three cycles, 43 (95.6%) proceeded to surgery, and 42 (93.3%) underwent R0 resection. Among the 43 patients who underwent surgical resection, the pathological responses of the primary tumor were JCGC grade 3, 2b, 2a, 1b, and 1a in 11 (25.6%), 7 (16.3%), 8 (18.6%), 4 (9.3%), and 13 (30.2%) patients, respectively. Adverse events during preoperative chemotherapy and surgery were previously reported [13]. Among the 42 patients who underwent R0 resection, 40 (95.2%) patients initiated postoperative chemotherapy with S-1, and 30 (71.4%) patients completed postoperative S-1 for 1 year after surgery. Grade 3 or 4 adverse events occurring in more than 5% of patients during postoperative chemotherapy included leukopenia, neutropenia, lymphopenia, and fatigue in 2 (5%), 2 (5%), 4 (10%), and 2 (5%) patients, respectively. No treatment-related deaths were observed during the 3-year follow-up period.

**Table 1:** Baseline characteristics of the 46 eligible patients

		(*n* = 46)
Age, years	Median (range)	68 (20–74)
Sex	Male	38 (83%)
	Female	8 (17%)
Macroscopic type	0	5 (11%)
	1	1 (2%)
	2	23 (50%)
	3	16 (35%)
	5	1 (2%)
Histological type	Differentiated	33 (72%)
	Undifferentiated	13 (28%)
Location of tumor	Upper third	14 (30%)
	Middle third	16 (35%)
	Lower third	16 (35%)
Esophageal involvement	Absent	42 (91%)
	Present	4 (9%)
cT category	T1b	2 (4%)
	T2	3 (7%)
	T3	8 (17%)
	T4	33 (72%)
cN category	N1	7 (15%)
	N2	28 (61%)
	N3	11 (24%)
cM category	M0	20 (43%)
	M1	26 (57%)
Node status	BN only	20 (43%)
	PAN only	17 (37%)
	Both BN and PAN	9 (20%)

BN, bulky node; PAN, para-aortic node

**Fig. 2:**
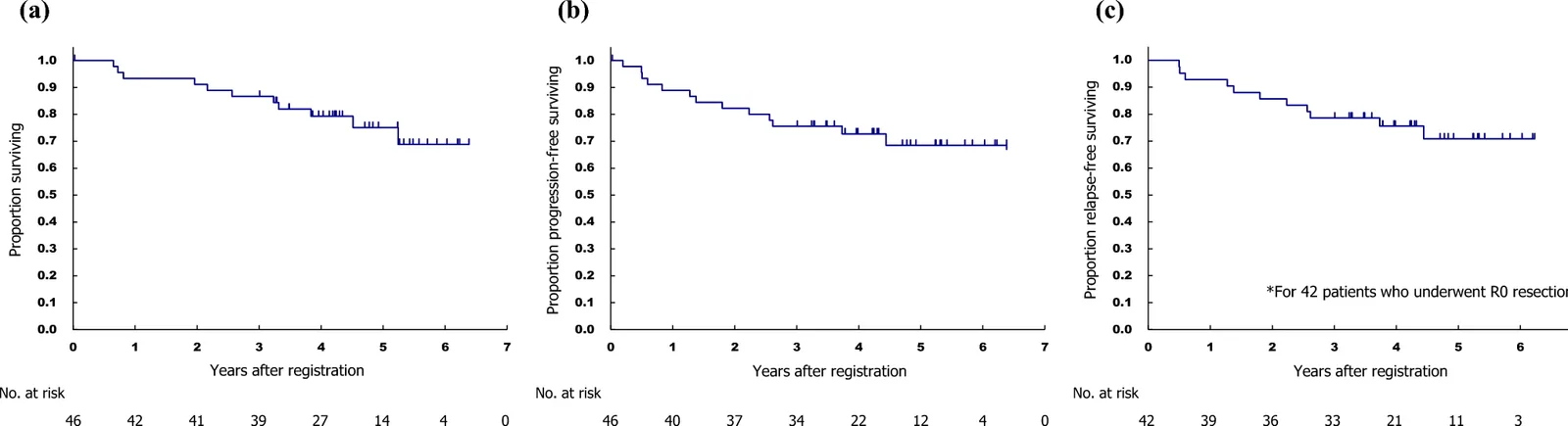
Kaplan–Meier curves for overall survival (**a**) and progression-free survival (**b**) in eligible patients and relapse-free survival (**c**) in patients who underwent R0 resection

At the data cutoff date of May 2025, the median follow-up duration for OS analysis was 4.2 years (interquartile range, 3.3–5.2 years). Among the 46 eligible patients, the 2- and 3-year OS were 91.1% (95% confidence interval [CI], 78.0–96.6%) and 86.7% (95% CI 72.7–93.8%), respectively (Fig. 2a). Among the 46 eligible patients, the 2- and 3-year PFS were 82.2% (95% CI 67.6–90.7%) and 75.6% (95% CI 60.2–85.6%), respectively (Fig. 2b). Causes of death included disease progression and other causes in 9 and 2 patients, respectively. Among the 42 patients who underwent R0 resection, the 2- and 3-year RFS were 85.7% (95% CI 70.9–93.3%) and 78.6% (95% CI 62.9–88.2%), respectively (Fig. 2c). Recurrence, which was observed in 12 patients, most commonly involved lymph nodes (n = 6), followed by liver (n = 5), and peritoneum (n = 4). Among the 26 patients diagnosed as PAN-positive at enrollment, only three patients were pathologically confirmed to have PAN metastasis after preoperative DOS chemotherapy. In contrast, none of the 20 patients diagnosed as PAN-negative at enrollment were found to have pathological PAN metastasis. One of three pathologically PAN-positive patients developed liver and lymph node recurrence 2.7 months after surgery and died 6.5 months after surgery, another was developed kidney, peritoneal, liver and PAN recurrence 3.9 months after surgery and died 5.5 months, whereas the other patient was alive without recurrence at the last follow-up evaluation 5 years after surgery.

**Fig. 3:**
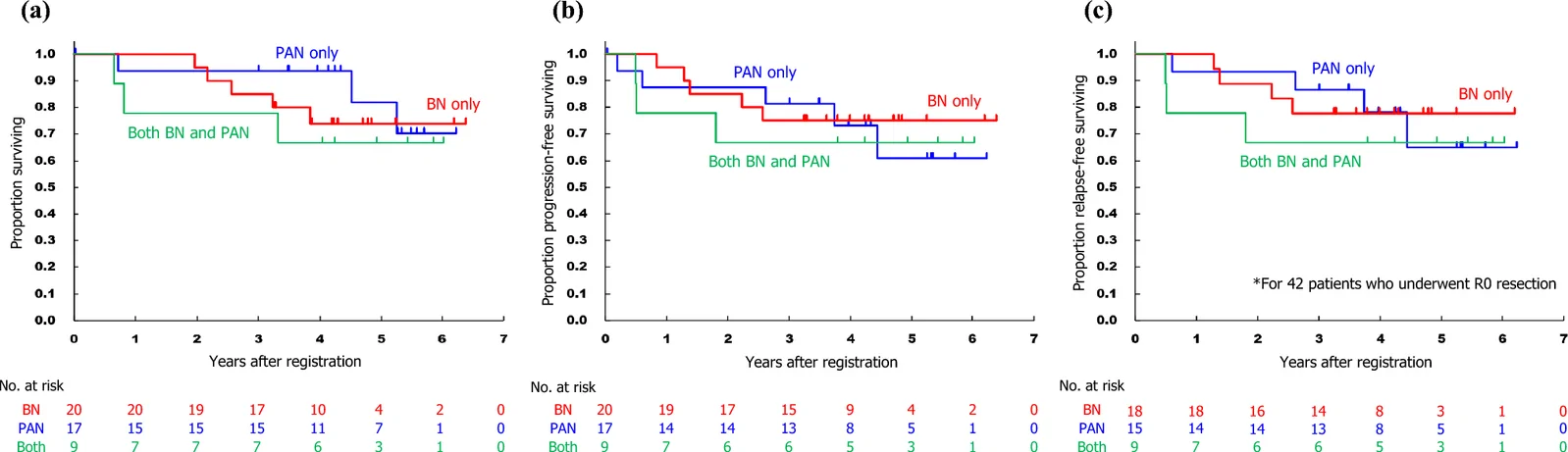
Kaplan–Meier curves for overall survival (**a**), progression-free survival (**b**), and relapse-free survival (**c**) according to clinical lymph node status BN, bulky node; PAN, para-aortic node

In subgroup analysis of the patients categorized according to the nodal status, the 3-year OS, PFS, and RFS were 85.0% (95% CI 60.4–94.9%), 75.0% (95% CI 50.0–88.7%), and 77.8% (95% CI 51.1–91.0%), respectively, in patients with only BN metastasis; 93.8% (95% CI 63.2–99.1%), 81.3% (95% CI 52.5–93.5%), and 86.7% (95% CI 56.4–96.5%), respectively, in those with only PAN metastasis; and 77.8% (95% CI 36.5–93.9%), 66.7% (95% CI 28.2–87.8%), and 66.7% (95% CI 28.2–87.8%), respectively, in patients with both BN and PAN metastases (Fig. 3).

**Fig. 4:**
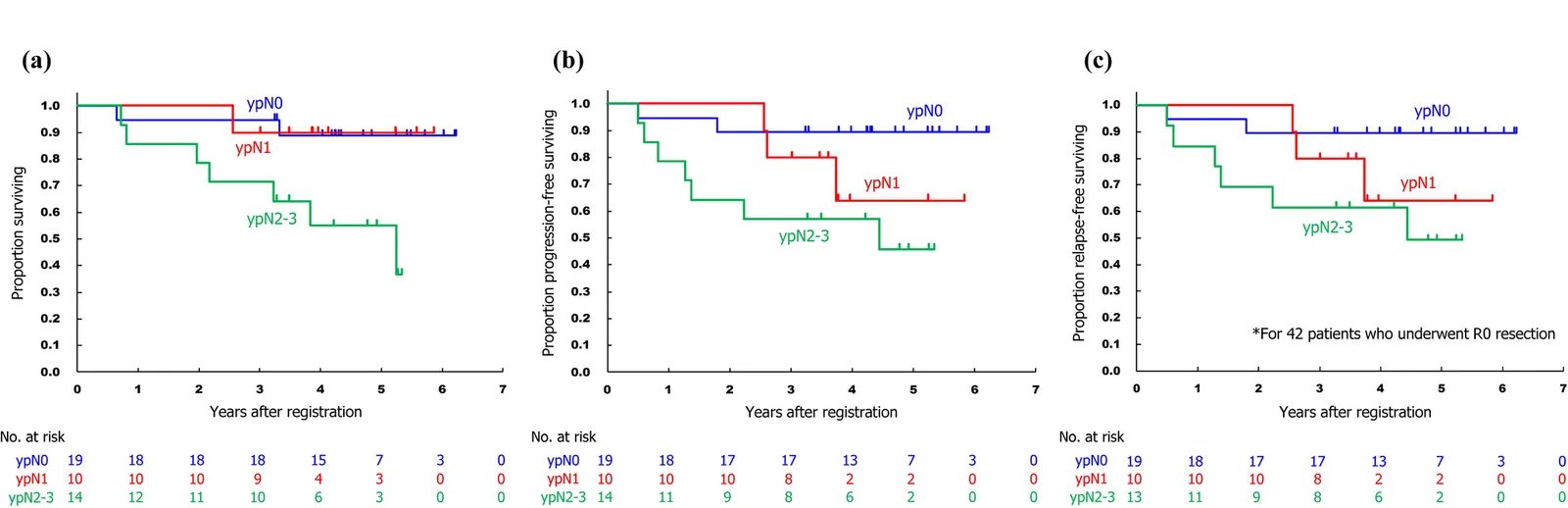
Kaplan–Meier curves for overall survival (**a**), progression-free survival (**b**), and relapse-free survival (**c**) stratified by ypN status

In subgroup analysis of the patients categorized stratified by ypN status, the 3-year OS, PFS, and RFS were 94.7% (95% CI 68.1–99.2%), 89.5% (95% CI 64.1–97.3%), and 89.5% (95% CI 64.1–97.3%), respectively, in patients with ypN0; 90.0% (95% CI 47.3–98.5%), 80.0% (95% CI 40.9–94.6%), and 80.0% (95% CI 40.9–94.6%), respectively, in those with ypN1; and 71.4% (95% CI 40.6–88.2%), 57.1% (95% CI 28.4–78.0%), and 61.5% (95% CI 30.8–81.8%), respectively, in those with ypN2-3 (Fig. 4).

**Fig. 5:**
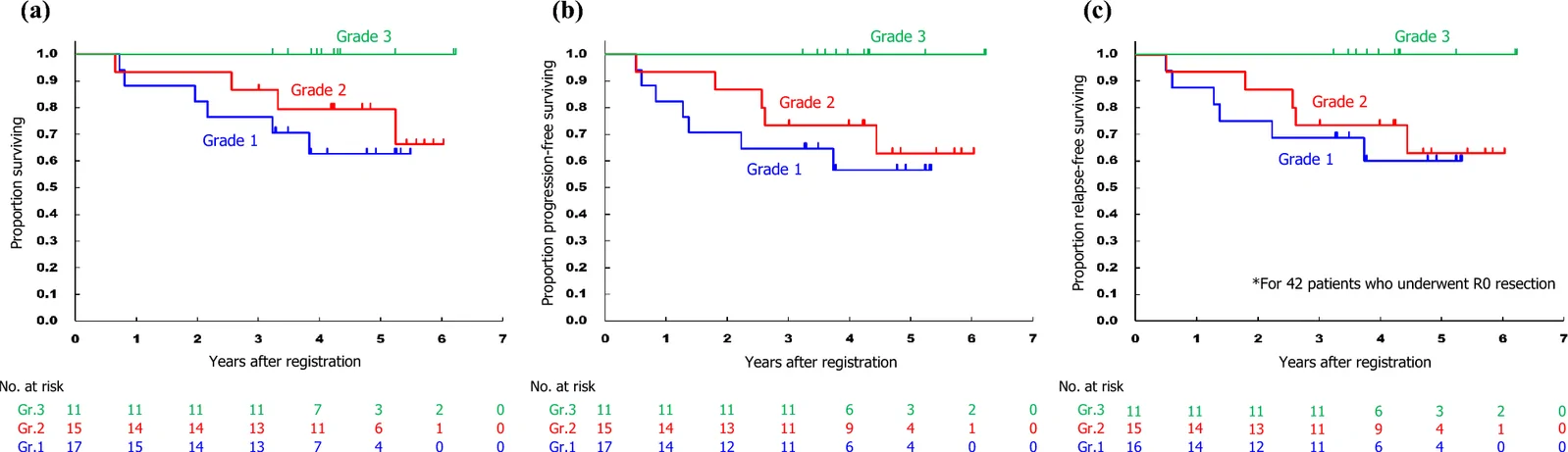
Kaplan–Meier curves for overall survival (**a**), progression-free survival (**b**), and relapse-free survival (**c**) according to pathological response Gr., grade based on the Japanese Classification of Gastric Carcinoma

In subgroup analysis of the patients categorized according to the pathological response based on the JCGC, the 3-year OS, PFS, and RFS were 100% (95% CI 100–100%), 100% (95% CI 100–100%), and 100% (95% CI 100–100%), respectively, in patients with JCGC grade 3 response; 86.7% (95% CI 56.4–96.5%), 73.3% (95% CI 43.6–89.1%), and 73.3% (95% CI 43.6–89.1%), respectively, in those with JCGC grade 2 response; and 76.5% (95% CI 48.8–90.4%), 64.7% (95% CI 37.7–82.3%), and 68.8% (95% CI 40.5–85.6%), respectively, in those with JCGC grade 1 response (Fig. 5).

## Discussion

The 3-year follow-up results of JCOG1704 demonstrated that preoperative chemotherapy with DOS achieved unprecedentedly favorable survival outcomes in gastric cancer patients with ELM. Preoperative DOS has been previously shown to yield favorable pathological responses with an acceptable toxicity profile [13]. Since 2000, the SCSG/JCOG have consistently conducted phase II trials using identical eligibility criteria to establish the optimal perioperative treatment for this patient population [5–7]. In this setting, JCOG1704 revealed that preoperative DOS followed by postoperative S-1 achieved markedly superior efficacy, with no treatment-related deaths, compared with previous treatment strategies (Table 2). While OS may be influenced by treatments administered after recurrence, PFS and RFS are not affected by post-recurrence therapies. In addition, both the surgical procedures and postoperative adjuvant therapy (1 year of S-1) were identical to those used in the previous JCOG1002 trial. Therefore, we believe that the favorable outcomes observed in OS, PFS, and RFS are largely attributable to differences in the preoperative treatment. Review of these JCOG trials involving the same patient population reveals major pRR as the surrogate endpoint that most accurately reflects long-term survival. Therefore, it would be reasonable to set major pRR as the primary endpoint in future phase II trials on preoperative chemotherapy for resectable gastric cancer, as was done in the present study.

**Table 2:** Summary of phase II trials conducted by the JCOG evaluating preoperative chemotherapy for gastric cancer with extensive lymph node metastasis

Regimen of preoperative chemotherapy	*n*	Major pRR (Grade ≥ 2)	pCR rate (Grade 3)	3-year OS	3-year RFS
irinotecan + cisplatin (JCOG0001)	55	11%	2%	27.0%	NE
cisplatin + S-1 (JCOG0405)	51	27%	2%	58.8%	50.0%
docetaxel + cisplatin + S-1 (JCOG1002)	52	35%	2%	62.7%	54.5%
docetaxel + oxaliplatin + S-1 (JCOG1704)	46	57%	24%	86.7%	78.6%

pRR, pathological response rate; pCR, pathological complete response; OS, overall survival; RFS, relapse-free survival; NE, not evaluated

Pathological response was assessed according to the Japanese Classification of Gastric Carcinoma

The markedly higher efficacy of DOS compared with cisplatin plus S-1 in JCOG0405 may be attributable to the additive effect of docetaxel, a key chemotherapeutic in the treatment of gastric cancer. In contrast, DOS exhibited substantially greater efficacy than docetaxel plus cisplatin plus S-1 (DCS) in JCOG1002, which may be explained by differences in overall drug exposure. Specifically, the total doses (dose intensities) of S-1 and docetaxel in the DCS regimen were 2240 mg/m^2^ (280 mg/m^2^/week) and 80 mg/m^2^ (10 mg/m^2^/week), respectively, which were considerably lower than those in the DOS regimen (3360 mg/m^2^ [373 mg/m^2^/week] and 120 mg/m^2^ [13 mg/m^2^/week], respectively). Notably, none of the 11 patients who achieved pCR after preoperative DOS had experienced recurrence at the last follow-up evaluation. Although longer follow-up is warranted, a nonoperative “watch-and-wait” strategy might become feasible in certain patients with gastric cancer, if a highly accurate method is available to predict pCR after highly active preoperative chemotherapy with substantial pathological effects, such as the DOS regimen.

The favorable outcomes observed with DOS in the present study are highly consistent with the results of the Korean phase III PRODIGY trial [9, 10], in which preoperative DOS followed by postoperative S-1 significantly improved OS and PFS compared with upfront surgery followed by postoperative S-1. Notably, in the forest plot analysis of OS, the hazard ratio decreased from 0.96 in the cN1 subgroup to 0.76 and 0.60 in the cN2 and cN3 subgroups, respectively, indicating that the benefit of preoperative DOS increased with greater lymph node involvement. These findings suggest that gastric cancer with ELM, targeted in JCOG1704, may have greater benefits from DOS therapy than gastric cancer with more limited lymph node metastasis. In contrast, perioperative FLOT (fluorouracil, leucovorin, oxaliplatin, and docetaxel) chemotherapy has been established as the standard treatment for resectable gastric cancer in Western countries, based on the results of the German phase III FLOT4 trial [15]. In FLOT4, the forest plot analysis of OS revealed hazard ratios of 0.642 and 0.806 in the clinically node-negative and clinically node-positive subgroups, respectively, suggesting that perioperative FLOT therapy may be less effective in patients with lymph node metastasis. Since both DOS and FLOT are triplet regimens containing oxaliplatin and docetaxel, the observed difference in efficacy based on nodal status may relate to differences between S-1 and fluorouracil plus leucovorin. Currently, the SCSG/JCOG are conducting a randomized phase II/III trial (JCOG2203) comparing upfront surgery, preoperative DOS, and preoperative FLOT in patients with resectable esophagogastric junction adenocarcinoma [16]; the trial is expected to clarify the comparative efficacy and safety of DOS and FLOT.

More recently, the global phase III MATTERHORN trial demonstrated that the addition of durvalumab to perioperative FLOT significantly improved OS and event-free survival in patients with resectable gastric or esophagogastric junction adenocarcinoma [17]. However, the pCR rate with durvalumab plus FLOT (D-FLOT) was 19% in MATTERHORN whereas the pCR rate with DOS was 24% in JCOG1704. Moreover, in MATTERHORN, which enrolled patients with cStage I–III disease according to the 14th edition of the JCGC, the 2-year OS and event-free survival with D-FLOT were 75.7% and 67.4%, respectively. In contrast, although 57% of the patients enrolled in JCOG1704 had cM1 disease, the 2-year OS and PFS were markedly higher (91.1% and 82.2%, respectively). These findings should be interpreted with caution, because no trial has directly compared the DOS and D-FLOT regimens; however, the available evidence does not provide sufficient justification to replace the current Japanese standard treatment regimens with D-FLOT, adopted in Western countries, for gastric cancer with ELM or resectable cStage II–III gastric cancer.

A major limitation of JCOG1704 is the relatively small sample size and the limited number of patients in subgroup analyses, owing to the focus on an uncommon subtype of gastric cancer. Although a phase III trial following JCOG1704 is ideal, such an approach is unrealistic in this small patient population. Moreover, the outcomes of the present study far exceeded those of prior trials; therefore, little disagreement exists within our community regarding the adoption of this approach as a provisional standard of care before proceeding to a phase III trial. Another limitation is that this study was conducted within the JCOG framework, comprising specialized high-volume centers in Japan with extensive experience in gastric cancer treatment. Therefore, caution should be exercised when generalizing these findings to other clinical settings, particularly those with less experience.

## Conclusions

Preoperative chemotherapy with DOS for marginally resectable gastric cancer with ELM showed unprecedentedly favorable 3-year survival results. The favorable survival outcomes observed in this study reflect the entire treatment strategy, including preoperative DOS chemotherapy, extended surgery with PAN dissection, and postoperative S-1 therapy, rather than the effect of chemotherapy alone. This treatment approach may facilitate the management of gastric cancer with ELM not as marginally resectable or cStage IV disease but as a conventionally resectable or cStage III gastric cancer. Our long-standing series of clinical trials in this population, which has continued since 2000, can therefore be considered complete.
